# A pilot project to increase trainee engagement using a social media platform – outcomes and lessons learnt

**DOI:** 10.1192/bjo.2021.393

**Published:** 2021-06-18

**Authors:** Amy Grimason, Shevonne Matheiken, Laura Somerville, Fiona Martin, Luke Baker, Kabir Garg, Aastha Sharma, Simon George Morris

**Affiliations:** 1 Northern Ireland Medical and Dental Training Agency, Belfast Health and Social Care Trust; 2 Northamtonshire Healthcare NHS Foundation Trust; 3 Northern Ireland Medical and Dental Training Agency, South Eastern Health and Social Care Trust; 4 Northern Ireland Medical and Dental Training Agency, Northern Health and Social Care Trust; 5 South London and Maudsley NHS Foundation Trust, Institute of Naval Medicine; 6 Oxleas NHS Foundation Trust, London; 7 Cambridgeshire and Peterborough NHS foundation trust; 8 Avon and Wiltshire Mental Health Partnership NHS Trust

## Abstract

**Aims:**

Engagement with members is an important issue for the Royal College of Psychiatrists (RCPsych) and an area for ongoing development. This is an issue that extends to Psychiatry trainees and the Psychiatric Trainees’ Committee (PTC) has adopted increasing engagement as one of its key aims. Divisional representatives in different areas of the UK had noted that trainees had limited knowledge of the PTC or its roles and projects both within the College and local areas. To improve this it was decided to pilot a project that established a social media platform for trainees to improve communication between the PTC, it's representatives and trainees. It was decided that Workplace (a professional version of Facebook) would be used. This had already been established in the Severn Deanery.

**Method:**

Northern Ireland (NI) and the East of England (EoE) deaneries were selected as pilot areas for the project. Preparation for the project included collaboration with trainees from the Severn deanery and meeting with the RCPsych Digital team. A scoping questionnaire was circulated to trainees in each deanery.

Following this, two closed groups were initiated on Workplace in August 2019 for Northern Ireland and East of England trainees.

**Result:**

Results from the survey sent prior to the social media pages being established indicated there was appetite among trainees for the project. The pages were established in July 2019. The pilot project was promoted by representatives.

In the initial phases, approximately 40% of trainees signed up. Information regarding college and local events, committee meeting updates and training opportunities was disseminated on the platform. There was evidence of early use by trainees outside of the representative group.

This however was not sustained and gradually use of the platform reduced over the pilot period, both in postings and membership. A further questionnaire circulated in July 2020 highlighted trainees’ concerns relating to the platform, including concerns around data protection and a high number of notifications associated with the Workplace medium. The ultimate impact on engagement was also felt to be minimal.

**Conclusion:**

Following feedback and increasing usage costs by Workplace, it was decided not to continue with a nationwide role out of the project. COVID-19 has seen the successful use of platforms such as Microsoft Teams and these may be considered in the future, given their integration with existing trust systems.

